# Supplementary material for: Machine Learning Accelerates Crystallization for Structure Determination
Source: Angew Chem Int Ed Engl. 2026 May 4;65(25):e1218503. doi: 10.1002/anie.1218503 (PMC13266958; doi:10.1002/anie.1218503)

checkCIF/PLATON report

Structure factors have been supplied for datablock(s) lcz023

THIS REPORT IS FOR GUIDANCE ONLY. IF USED AS PART OF A REVIEW PROCEDURE FOR PUBLICATION, IT SHOULD NOT REPLACE THE EXPERTISE OF AN EXPERIENCED CRYSTALLOGRAPHIC REFEREE.

No syntax errors found. CIF dictionary Interpreting this report

Datablock: lcz023

Bond precision:	C-C = 0.0174 A		Wavelength=1.54184
Cell:	a=8.3525(5)	b=22.6623(11)	c=17.9457(11)
	alpha=90	beta=96.182(5)	gamma=90
Temperature:	100 K		

	Calculated	Reported
Volume	3377.1(3)	3377.1(3)
Space group	P 21/n	P 1 21/n 1
Hall group	-P 2yn	-P 2yn
Moiety formula	C28 H15 Ag3 F18 N6 O2	C15 H3 Ag3 F18 N6, C9.5 H8.5 O1.333, C3.5 H3.5 O0.667
Sum formula	C28 H15 Ag3 F18 N6 O2	C28 H15 Ag3 F18 N6 O2
Mr	1133.07	1133.07
Dx,g cm-3	2.229	2.229
Z	4	4
Mu (mm-1)	15.130	15.130
F000	2176.0	2176.0
F000’	2186.42	
h,k,lmax	10,28,22	10,27,21
Nref	6729	6506
Tmin,Tmax	0.104,0.163	0.501,1.000
Tmin’	0.022	

Correction method= # Reported T Limits: Tmin=0.501 Tmax=1.000
AbsCorr = MULTI-SCAN

Data completeness= 0.967 Theta(max)= 72.830

R(reflections)= 0.1054(4792) wR2(reflections)= 0.2927(6506)

S = 1.123 Npar= 761

The following ALERTS were generated. Each ALERT has the format
test-name_ALERT_alert-type_alert-level.
Click on the hyperlinks for more details of the test.

Alert level C

```
PLAT042_ALERT_1_C Calc. and Reported MoietyFormula Strings  Differ     Please Check
              Calc: C28 H15 Ag3 F18 N6 O2
              Rep.: C15 H3 Ag3 F18 N6, C9.5 H8.5 O1.333, C3.5 H3.5
                     O0.667
PLAT082_ALERT_2_C High R1 Value ..................................       0.11 Report
PLAT084_ALERT_3_C High wR2 Value (i.e. > 0.25) ...................       0.29 Report
PLAT088_ALERT_3_C Poor Data / Parameter Ratio ....................       8.55 Note
PLAT215_ALERT_3_C Disordered F4        has ADP max/min Ratio .....        3.2 Note
PLAT215_ALERT_3_C Disordered F4#       has ADP max/min Ratio .....        3.1 Note
PLAT215_ALERT_3_C Disordered F9        has ADP max/min Ratio .....        3.7 Note
PLAT215_ALERT_3_C Disordered F49       has ADP max/min Ratio .....        3.5 Note
PLAT220_ALERT_2_C NonSolvent   Resd 1  C   Ueq(max)/Ueq(min) Range        5.5 Ratio
PLAT220_ALERT_2_C NonSolvent   Resd 1 Ag   Ueq(max)/Ueq(min) Range        3.8 Ratio
PLAT222_ALERT_3_C NonSolvent Resd 1  H   Uiso(max)/Uiso(min) Range        5.3 Ratio
PLAT234_ALERT_4_C Large Hirshfeld Difference Ag       --N00G     .       0.18 Ang.
PLAT234_ALERT_4_C Large Hirshfeld Difference Ag1      --N005     .       0.17 Ang.
PLAT234_ALERT_4_C Large Hirshfeld Difference Ag02     --N00A     .       0.16 Ang.
PLAT234_ALERT_4_C Large Hirshfeld Difference F009     --C01G     .       0.20 Ang.
PLAT234_ALERT_4_C Large Hirshfeld Difference F00J     --C01G     .       0.20 Ang.
PLAT234_ALERT_4_C Large Hirshfeld Difference F12      --C9       .       0.20 Ang.
PLAT234_ALERT_4_C Large Hirshfeld Difference N00A     --C013     .       0.25 Ang.
PLAT234_ALERT_4_C Large Hirshfeld Difference C00Y     --C013     .       0.18 Ang.
PLAT234_ALERT_4_C Large Hirshfeld Difference C013     --C01M     .       0.22 Ang.
PLAT234_ALERT_4_C Large Hirshfeld Difference C00V     --C011     .       0.20 Ang.
PLAT234_ALERT_4_C Large Hirshfeld Difference C01F     --C00R     .       0.19 Ang.
PLAT234_ALERT_4_C Large Hirshfeld Difference C00R     --C01H     .       0.23 Ang.
PLAT234_ALERT_4_C Large Hirshfeld Difference C019     --C012     .       0.23 Ang.
PLAT342_ALERT_3_C Low Bond Precision on  C-C Bonds ...............    0.01737 Ang.
PLAT362_ALERT_2_C Short  C(sp3)-C(sp2) Bond  C01C     - C9       .       1.41 Ang.
PLAT362_ALERT_2_C Short  C(sp3)-C(sp2) Bond  C00U     - C00P     .       1.40 Ang.
PLAT906_ALERT_3_C Large K Value in the Analysis of Variance ......     25.110 Check
PLAT906_ALERT_3_C Large K Value in the Analysis of Variance ......      5.439 Check
PLAT906_ALERT_3_C Large K Value in the Analysis of Variance ......      2.512 Check
PLAT911_ALERT_3_C Missing FCF Refl Between Thmin & STh/L=    0.600         13 Report
                4 24  1,  -1 27  1, -10  0  2,   2 22  2,   5 23  2, -10  1  3,
               -5 23  4,  -5 23  5,  -4 24  5,  -2 26  5,  -6 20  8,  -9  6 10,
                1  0 21,
PLAT971_ALERT_2_C Check Calcd Resid. Dens.  1.75Ang From F2             1.88 eA-3
```

Alert level G

```
PLAT002_ALERT_2_G Number of Distance or Angle Restraints on AtSite         48 Note
PLAT003_ALERT_2_G Number of Uiso or U(i,j)  Restrained non-H-Atoms         60 Report
PLAT004_ALERT_5_G Polymeric Structure Found with Maximum Dimension          1 Info
PLAT072_ALERT_2_G SHELXL First  Parameter in WGHT  Unusually Large       0.12 Report
PLAT083_ALERT_2_G SHELXL Second Parameter in WGHT  Unusually Large      51.22 Why ?
PLAT172_ALERT_4_G The CIF-Embedded .res File Contains DFIX Records         40 Report
PLAT174_ALERT_4_G The CIF-Embedded .res File Contains FLAT Records          1 Report
PLAT176_ALERT_4_G The CIF-Embedded .res File Contains SADI Records          8 Report
PLAT177_ALERT_4_G The CIF-Embedded .res File Contains DELU Records          9 Report
PLAT178_ALERT_4_G The CIF-Embedded .res File Contains SIMU Records         17 Report
PLAT186_ALERT_4_G The CIF-Embedded .res File Contains ISOR Records         13 Report
PLAT188_ALERT_3_G A Non-default SIMU Restraint Value has been used     0.0100 Report
PLAT188_ALERT_3_G A Non-default SIMU Restraint Value has been used     0.0100 Report
PLAT188_ALERT_3_G A Non-default SIMU Restraint Value has been used     0.0100 Report
PLAT188_ALERT_3_G A Non-default SIMU Restraint Value has been used     0.0100 Report
PLAT188_ALERT_3_G A Non-default SIMU Restraint Value has been used     0.0100 Report
PLAT188_ALERT_3_G A Non-default SIMU Restraint Value has been used     0.0100 Report
PLAT188_ALERT_3_G A Non-default SIMU Restraint Value has been used     0.0100 Report
PLAT188_ALERT_3_G A Non-default SIMU Restraint Value has been used     0.0100 Report
PLAT188_ALERT_3_G A Non-default SIMU Restraint Value has been used     0.0100 Report
PLAT188_ALERT_3_G A Non-default SIMU Restraint Value has been used     0.0100 Report
PLAT188_ALERT_3_G A Non-default SIMU Restraint Value has been used     0.0100 Report
PLAT232_ALERT_2_G Hirshfeld Test Diff (M-X)  Ag01     --N005     .        5.3 s.u.
PLAT232_ALERT_2_G Hirshfeld Test Diff (M-X)  Ag03     --N00G     .        9.0 s.u.
PLAT242_ALERT_2_G Low    'MainMol' Ueq as Compared to Neighbors of       C00U Check
PLAT242_ALERT_2_G Low    'MainMol' Ueq as Compared to Neighbors of       C016 Check
PLAT242_ALERT_2_G Low    'MainMol' Ueq as Compared to Neighbors of         C9 Check
PLAT242_ALERT_2_G Low    'MainMol' Ueq as Compared to Neighbors of       C01G Check
PLAT299_ALERT_4_G Atom Site Occupancy Constrained at ............         0.5 Check
              F1      F006    F00C    F4      F00D    F5      F00E    F6
              F00H    F7      F00I    F8      F9      F00O    F01B    F01L
              F14     F17     F20     F22     F24     F39     F49     F83
              N00F    N008    N12     N25     C5      C10     C010    C83
PLAT300_ALERT_4_G Atom Site Occupancy of O1         Constrained at     0.6667 Check
PLAT300_ALERT_4_G Atom Site Occupancy of O4         Constrained at     0.6667 Check
PLAT300_ALERT_4_G Atom Site Occupancy of O18        Constrained at     0.3333 Check
PLAT300_ALERT_4_G Atom Site Occupancy of O19        Constrained at     0.3333 Check
PLAT300_ALERT_4_G Atom Site Occupancy of C2         Constrained at       0.75 Check
PLAT300_ALERT_4_G Atom Site Occupancy of C3         Constrained at     0.6667 Check
PLAT300_ALERT_4_G Atom Site Occupancy of C4         Constrained at     0.6667 Check
PLAT300_ALERT_4_G Atom Site Occupancy of C01A       Constrained at       0.75 Check
PLAT300_ALERT_4_G Atom Site Occupancy of C00W       Constrained at       0.75 Check
PLAT300_ALERT_4_G Atom Site Occupancy of C01F       Constrained at       0.75 Check
PLAT300_ALERT_4_G Atom Site Occupancy of C00R       Constrained at       0.75 Check
PLAT300_ALERT_4_G Atom Site Occupancy of C01H       Constrained at       0.75 Check
PLAT300_ALERT_4_G Atom Site Occupancy of C018       Constrained at       0.75 Check
PLAT300_ALERT_4_G Atom Site Occupancy of C01I       Constrained at       0.75 Check
PLAT300_ALERT_4_G Atom Site Occupancy of C16        Constrained at     0.6667 Check
PLAT300_ALERT_4_G Atom Site Occupancy of C019       Constrained at       0.75 Check
PLAT300_ALERT_4_G Atom Site Occupancy of C012       Constrained at       0.75 Check
PLAT300_ALERT_4_G Atom Site Occupancy of C014       Constrained at       0.75 Check
PLAT300_ALERT_4_G Atom Site Occupancy of C33        Constrained at     0.6667 Check
PLAT300_ALERT_4_G Atom Site Occupancy of C1         Constrained at       0.25 Check
PLAT300_ALERT_4_G Atom Site Occupancy of C6         Constrained at     0.3333 Check
PLAT300_ALERT_4_G Atom Site Occupancy of C7         Constrained at       0.25 Check
PLAT300_ALERT_4_G Atom Site Occupancy of C12        Constrained at       0.25 Check
```

```
PLAT300_ALERT_4_G Atom Site Occupancy of C17        Constrained at     0.3333 Check
PLAT300_ALERT_4_G Atom Site Occupancy of C01O       Constrained at       0.25 Check
PLAT300_ALERT_4_G Atom Site Occupancy of C017       Constrained at       0.25 Check
PLAT300_ALERT_4_G Atom Site Occupancy of C24        Constrained at     0.3333 Check
PLAT300_ALERT_4_G Atom Site Occupancy of C25        Constrained at       0.25 Check
PLAT300_ALERT_4_G Atom Site Occupancy of C26        Constrained at       0.25 Check
PLAT300_ALERT_4_G Atom Site Occupancy of C28        Constrained at       0.25 Check
PLAT300_ALERT_4_G Atom Site Occupancy of C29        Constrained at       0.25 Check
PLAT300_ALERT_4_G Atom Site Occupancy of C30        Constrained at       0.25 Check
PLAT300_ALERT_4_G Atom Site Occupancy of C35        Constrained at     0.3333 Check
PLAT300_ALERT_4_G Atom Site Occupancy of C63        Constrained at       0.25 Check
PLAT300_ALERT_4_G Atom Site Occupancy of H00W       Constrained at       0.75 Check
PLAT300_ALERT_4_G Atom Site Occupancy of H3A        Constrained at     0.6667 Check
PLAT300_ALERT_4_G Atom Site Occupancy of H3B        Constrained at     0.6667 Check
PLAT300_ALERT_4_G Atom Site Occupancy of H3C        Constrained at     0.6667 Check
PLAT300_ALERT_4_G Atom Site Occupancy of H01F       Constrained at       0.75 Check
PLAT300_ALERT_4_G Atom Site Occupancy of H01I       Constrained at       0.75 Check
PLAT300_ALERT_4_G Atom Site Occupancy of H019       Constrained at       0.75 Check
PLAT300_ALERT_4_G Atom Site Occupancy of H012       Constrained at       0.75 Check
PLAT300_ALERT_4_G Atom Site Occupancy of H014       Constrained at       0.75 Check
PLAT300_ALERT_4_G Atom Site Occupancy of H16        Constrained at     0.6667 Check
PLAT300_ALERT_4_G Atom Site Occupancy of H33A       Constrained at     0.6667 Check
PLAT300_ALERT_4_G Atom Site Occupancy of H33B       Constrained at     0.6667 Check
PLAT300_ALERT_4_G Atom Site Occupancy of H33C       Constrained at     0.6667 Check
PLAT300_ALERT_4_G Atom Site Occupancy of H6         Constrained at     0.3333 Check
PLAT300_ALERT_4_G Atom Site Occupancy of H01O       Constrained at       0.25 Check
PLAT300_ALERT_4_G Atom Site Occupancy of H24A       Constrained at     0.3333 Check
PLAT300_ALERT_4_G Atom Site Occupancy of H24B       Constrained at     0.3333 Check
PLAT300_ALERT_4_G Atom Site Occupancy of H24C       Constrained at     0.3333 Check
PLAT300_ALERT_4_G Atom Site Occupancy of H25        Constrained at       0.25 Check
PLAT300_ALERT_4_G Atom Site Occupancy of H28        Constrained at       0.25 Check
PLAT300_ALERT_4_G Atom Site Occupancy of H29        Constrained at       0.25 Check
PLAT300_ALERT_4_G Atom Site Occupancy of H30        Constrained at       0.25 Check
PLAT300_ALERT_4_G Atom Site Occupancy of H35A       Constrained at     0.3333 Check
PLAT300_ALERT_4_G Atom Site Occupancy of H35B       Constrained at     0.3333 Check
PLAT300_ALERT_4_G Atom Site Occupancy of H35C       Constrained at     0.3333 Check
PLAT300_ALERT_4_G Atom Site Occupancy of H63        Constrained at       0.25 Check
PLAT301_ALERT_3_G Main Residue  Disorder .............(Resd    1)        63% Note
PLAT432_ALERT_2_G Short Inter X...Y Contact  F009     ..C28      .       2.96 Ang.
                                            1-x,1-y,1-z  =      3_666 Check
PLAT432_ALERT_2_G Short Inter X...Y Contact  F6       ..C011     .       2.88 Ang.
                                      3/2-x,1/2+y,3/2-z  =      2_656 Check
PLAT434_ALERT_2_G Short Inter HL..HL Contact F009     ..F24      .       2.76 Ang.
                                            1-x,1-y,1-z  =      3_666 Check
PLAT720_ALERT_4_G Number of Unusual/Non-Standard Labels ..........         53 Note
              Ag01    Ag02    Ag03    F006    F009    F00C    F00D    F00E
              N00G    C01C    C00Y    H00Y    C013    N00A    F00H    F00I
              F00J    F00O    F00Q    C00U    C00V    C011    H011    C00P
              N005    N004    C016    C01A    C00W    H00W    C01F    H01F
              C00R    C01H    C018    C01I    H01I    C019    H019    C012
              H012    C014    H014    F01B    C01G    F01L    C01M    C01O
              H01O    C017    N00F    N008    C010
PLAT780_ALERT_1_G Coordinates do not Form a Properly Connected Set     Please Do !
PLAT811_ALERT_5_G No ADDSYM Analysis: Too Many Excluded Atoms ....          ! Info
PLAT860_ALERT_3_G Number of Least-Squares Restraints ............         508 Note
PLAT883_ALERT_1_G Absent Datum for _atom_sites_solution_primary ..     Please Do !
PLAT910_ALERT_3_G Missing FCF Reflection(s) Below Theta(Min)[Deg]=       3.90 Note
```

```
               0  1  1,
PLAT912_ALERT_4_G Missing # of FCF Reflections Above STh/L=  0.600        196 Note
PLAT933_ALERT_2_G Number of HKL-OMIT Records in Embedded .res File          4 Note
              -6 20  8,  -4 24  5,  -4 25  3,  -3 26  2,
PLAT941_ALERT_3_G Average HKL Measurement Multiplicity ..........         3.8 Low
PLAT969_ALERT_5_G The 'Henn et al.' R-Factor-gap value ..........       5.957 Note
              Predicted wR2: Based on SigI**2  4.91 or SHELX Weight 26.06
PLAT978_ALERT_2_G Number C-C Bonds with Positive Residual Density.          2 Info
```

```
   0 ALERT level A = Most likely a serious problem - resolve or explain
   0 ALERT level B = A potentially serious problem, consider carefully
  32 ALERT level C = Check. Ensure it is not caused by an omission or oversight
 104 ALERT level G = General information/check it is not something unexpected

   3 ALERT type 1 CIF construction/syntax error, inconsistent or missing data
  21 ALERT type 2 Indicator that the structure model may be wrong or deficient
  27 ALERT type 3 Indicator that the structure quality may be low
  82 ALERT type 4 Improvement, methodology, query or suggestion
   3 ALERT type 5 Informative message, check
```

Validation response form

Please find below a validation response form (VRF) that can be filled in and pasted into your CIF.

```
# start Validation Reply Form
_vrf_PLAT042_lcz023
;
PROBLEM: Calc. and Reported MoietyFormula Strings  Differ     Please Check
RESPONSE: ...
;
_vrf_PLAT082_lcz023
;
PROBLEM: High R1 Value .................................       0.11 Report
RESPONSE: ...
;
_vrf_PLAT084_lcz023
;
PROBLEM: High wR2 Value (i.e. > 0.25) ..................       0.29 Report
RESPONSE: ...
;
_vrf_PLAT088_lcz023
;
PROBLEM: Poor Data / Parameter Ratio ...................       8.55 Note
RESPONSE: ...
;
_vrf_PLAT215_lcz023
;
PROBLEM: Disordered F4        has ADP max/min Ratio .....        3.2 Note
RESPONSE: ...
;
_vrf_PLAT220_lcz023
;
PROBLEM: NonSolvent   Resd 1  C   Ueq(max)/Ueq(min) Range        5.5 Ratio
RESPONSE: ...
;
```

```
_vrf_PLAT222_lcz023
;
PROBLEM: NonSolvent Resd 1  H   Uiso(max)/Uiso(min) Range        5.3 Ratio
RESPONSE: ...
;
_vrf_PLAT234_lcz023
;
PROBLEM: Large Hirshfeld Difference Ag       --N00G     .       0.18 Ang.
RESPONSE: ...
;
_vrf_PLAT342_lcz023
;
PROBLEM: Low Bond Precision on  C-C Bonds ...............    0.01737 Ang.
RESPONSE: ...
;
_vrf_PLAT362_lcz023
;
PROBLEM: Short  C(sp3)-C(sp2) Bond  C01C     - C9       .       1.41 Ang.
RESPONSE: ...
;
_vrf_PLAT906_lcz023
;
PROBLEM: Large K Value in the Analysis of Variance ......     25.110 Check
RESPONSE: ...
;
_vrf_PLAT911_lcz023
;
PROBLEM: Missing FCF Refl Between Thmin & STh/L=    0.600         13 Report
RESPONSE: ...
;
_vrf_PLAT971_lcz023
;
PROBLEM: Check Calcd Resid. Dens.  1.75Ang From F2             1.88 eA-3
RESPONSE: ...
;
# end Validation Reply Form
```

It is advisable to attempt to resolve as many as possible of the alerts in all categories. Often the minor alerts point to easily fixed oversights, errors and omissions in your CIF or refinement strategy, so attention to these fine details can be worthwhile. It is up to the individual to critically assess their own results and, if necessary, seek expert advice.

PLATON version of 17/09/2025; check.def file version of 17/09/2025

---

duplicate check

No duplication found

Datablock lcz023 - ellipsoid plot

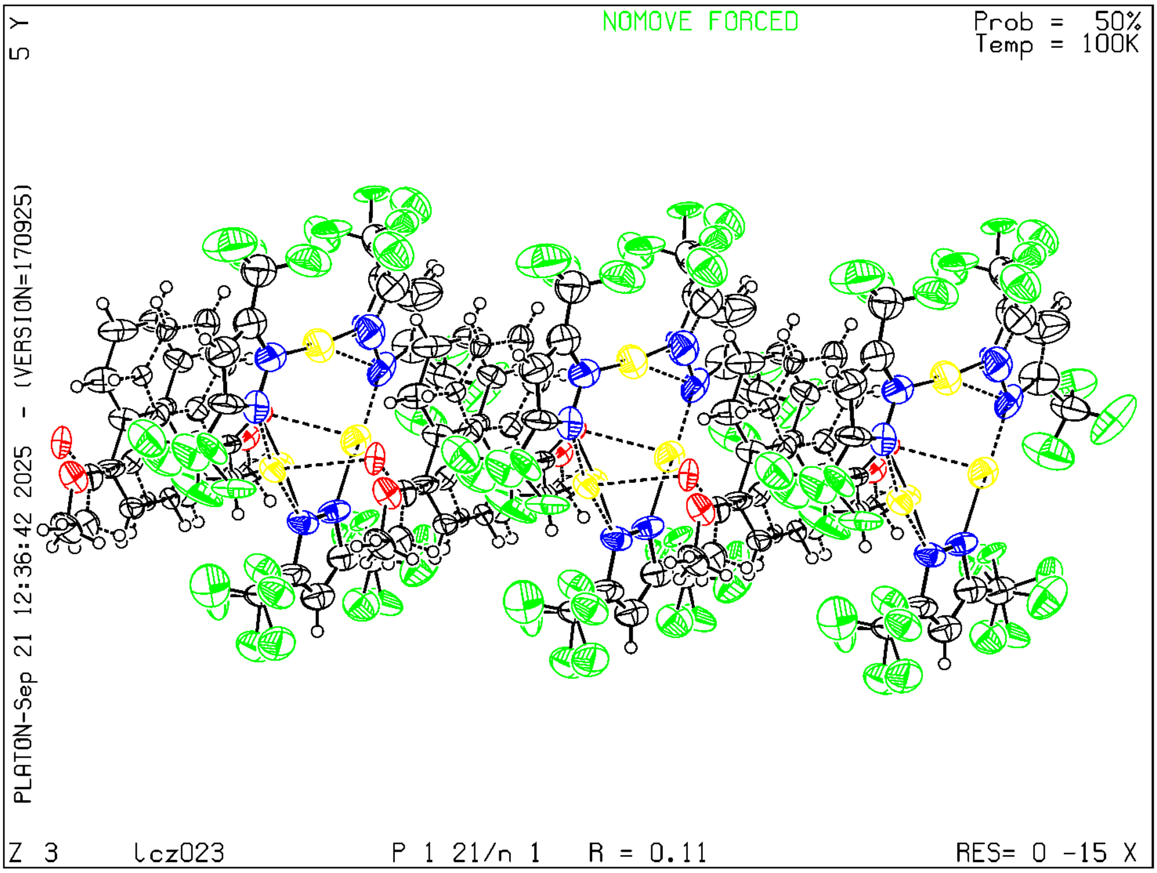

Supplement: Supplementary file 2 — Supporting File 2: anie72462‐sup‐0002‐DataFile.zip. [file ANIE-65-e1218503-s002.zip › supplementary_materials_crystallographic_data submit.zip!/cif_and_checkcif(1-114)/Ag3Pz3_23/Ag3Pz3_23_checkcif.pdf]
